# P26 Phenotypic and genotypic characterization of antimicrobial resistance in *Clostridioides difficile* in North Wales

**DOI:** 10.1093/jacamr/dlac004.025

**Published:** 2022-02-16

**Authors:** R. Thomas, B. Coupe, M. Bull, S. Copsey, T. Morris, M. Perry, C. Morgan

## Abstract

**Background:**

*Clostridioides difficile* poses a major infection control challenge and remains a huge burden to our healthcare system. The emergence of hypervirulent strains of *C. difficile* has highlighted the need for monitoring antimicrobial resistance (AMR) and continual epidemiological vigilance. Agar dilution is the current gold standard method for antimicrobial susceptibility testing of *C. difficile* and the method routinely utilized in the Public Health Wales (PHW) UK Anaerobe Reference Unit (UKARU) at the University Hospital of Wales, Cardiff. However, this method is laborious and time consuming. Meanwhile, the emergence of WGS introduces the possibility of acquiring rapid genotypic information in terms of AMR.

**Methods:**

In this research project, isolates obtained from faecal samples submitted across the Betsi Cadwalader health board (BCUHB) that were either GDH or PCR positive were sequenced as part of the *C. difficile* Genomic Sequencing and Typing (DIGEST) pilot project performed at the PHW Pathogen Genomics Unit (PenGU) in Cardiff.

**Results:**

Results highlights include the finding that 93% of the isolates analysed for the presence of AMR genes were found to have the *cdeA* gene associated with conferring resistance to fluoroquinolones such as ciprofloxacin. Other common genes observed included *blaCDD-1* and *blaCDD-2*, associated with β-lactam resistance.

**Conclusions:**

While further work must be done to link genotypic characterization with phenotype, this study provides a valuable insight into AMR mechanisms in *C. difficile* and may in future become an important tool providing early indication of shift in *C. difficile* epidemiology or the emergence of a new hypervirulent and MDR strain.

